# Is it how they walk? Biomechanics in diabetic peripheral neuropathy: a review of the literature

**DOI:** 10.1186/1757-1146-6-S1-P4

**Published:** 2013-05-31

**Authors:** Malindu Fernando, Robert Crowther, Peter Lazzarini, Kunwarjit Sangla, Margaret Cunningham, Jonathan Golledge

**Affiliations:** 1 Vascular Biology Unit, Queensland Research Centre for Peripheral Vascular Disease, School of Medicine and Dentistry, James Cook University, Townsville, Queensland, 4814, Australia; 2 Movement Analysis Laboratory, Institute of Sports and Exercise Science, James Cook University, Townsville, Queensland, 4814, Australia; 3 Allied Health Research Collaborative, Metro North Hospital & Health Service, Queensland Health, Brisbane, Queensland, 4515, Australia; 4 School of Clinical Sciences, Queensland University of Technology, Brisbane, Queensland, 4000, Australia; 5 Department of Internal Medicine, Townsville Hospital, Townsville, Queensland, 4814, Australia; 6 Department of Podiatry, Townsville Community Health Services, Kirwan Health Campus, Townsville, Queensland, 4817, Australia

## Background

Diabetic peripheral neuropathy (DPN) affects the sensory, motor, and autonomic nervous system. The biomechanical changes resulting from DPN may translate to increased plantar pressures in the foot, which contributes to the pathogenesis and development of foot ulcers. This review aims to investigate the existing biomechanical literature associated with gait, dynamic electromyography and plantar pressure of patients with DPN.

## Methods

Electronic databases (MEDLINE, CINAHL, PubMed, Scopus and Google Scholar) were searched for papers reporting observational studies on patients with DPN in gait, dynamic electromyography or plantar pressure. Exclusion criteria were papers investigating children, interventional studies or studies published prior to 2000.

## Results

Twenty-five papers met the inclusion criteria and were reviewed. Overall there were disparities between studies due to methodological differences in reporting such as the disease duration and degree of neuropathy of participants. DPN subjects walked slower, with smaller stride length and reduced knee extension and active ankle plantar/dorsiflexion compared to healthy and diabetes controls. Dynamic electromyography studies suggested an early activation of lateral gastrocnemius, whilst findings in the tibialis anterior and vastus lateralis muscles were inconsistent. Markedly elevated forefoot peak plantar pressures (PPP) were observed in those with a history of ulceration.

## Conclusion

This review suggests marked biomechanical (gait, electromyography and plantar pressure) variation in DPN patients compared to controls. Studies investigating kinematic (description of movement) variables of the foot are lacking and further studies are needed. It is recommended that future DPN biomechanical studies should document the duration and degree of DPN.

